# Supplementary material for: Synthesis of LiDAR-Detectable True Black Core/Shell Nanomaterial and Its Practical Use in LiDAR Applications
Source: Nanomaterials (Basel). 2022 Oct 20;12(20):3689. doi: 10.3390/nano12203689 (PMC9610704; doi:10.3390/nano12203689)
Supplement: Supplementary file 1 [file nanomaterials-12-03689-s001.zip › nanomaterials-1895036-supplementary.pdf]

nanomaterials

Supplementary Materials

Synthesis of LiDAR-Detectable True Black Core/Shell Nanomaterial and Its Practical Use in LiDAR Applications

Suk Jekal [1,†], Jiwon Kim [1,†], Dong-Hyun Kim [1], Jungchul Noh [2], Min-Jeong Kim [1], Ha-Yeong Kim [1], Min-Sang Kim [1], Won-Chun Oh [3] and Chang-Min Yoon [1,*]

[1] Department of Chemical and Biological Engineering, Hanbat National University, Yuseong-gu, Daejeon 34158, Korea
[2] McKetta Department of Chemical Engineering and Texas Material Institute, The University of Texas at Austin, Austin, TX 78712, USA
[3] Department of Advanced Materials Science and Engineering, Hanseo University, Seosan-si 31962, Korea
* Correspondence: cmyoon4321@hanbat.ac.kr; Tel.: +82-42-821-1528; Fax: +82-42-821-1593
† These authors contributed equally to this work.

Table S1. Elemental compositions of 100 nm SiO_2 nanoparticles, 130 nm ST CSNs, and 140 nm SBT CSNs [a.]

Material	Elemental composition (At.%)			
	Si	Ti	O	Ti/Si ratio [b]
100 nm SiO_2	27.19	-	72.81	-
130 nm ST CSNs	19.85	5.51	74.64	0.36
140 nm SBT CSNs	18.67	6.02	75.31	0.31

[a] Elemental composition of samples was obtained using the EDS mode installed in the TEM equipment, excluding all other elements except Si, Ti, and O (beam current: 10.0 μA, accelerating voltage: 10.0 kV).

[b] Ti/Si ratio were only determined for ST CSNs and SBT CSNs, since core SiO2 nanoparticles contain no Ti element.
